# Large Language Model−Based Chatbot vs Surgeon-Generated Informed Consent Documentation for Common Procedures

**DOI:** 10.1001/jamanetworkopen.2023.36997

**Published:** 2023-10-09

**Authors:** Hannah Decker, Karen Trang, Joel Ramirez, Alexis Colley, Logan Pierce, Melissa Coleman, Tasce Bongiovanni, Genevieve B. Melton, Elizabeth Wick

**Affiliations:** 1 Department of Surgery, University of California, San Francisco; 2 Department of Medicine, University of California, San Francisco; 3 Department of Surgery, Institute for Health Informatics, and Center for Learning Health System Sciences, University of Minnesota, Minneapolis

## Abstract

**Importance:**

Informed consent is a critical component of patient care before invasive procedures, yet it is frequently inadequate. Electronic consent forms have the potential to facilitate patient comprehension if they provide information that is readable, accurate, and complete; it is not known if large language model (LLM)-based chatbots may improve informed consent documentation by generating accurate and complete information that is easily understood by patients.

**Objective:**

To compare the readability, accuracy, and completeness of LLM-based chatbot- vs surgeon-generated information on the risks, benefits, and alternatives (RBAs) of common surgical procedures.

**Design, Setting, and Participants:**

This cross-sectional study compared randomly selected surgeon-generated RBAs used in signed electronic consent forms at an academic referral center in San Francisco with LLM-based chatbot-generated (ChatGPT-3.5, OpenAI) RBAs for 6 surgical procedures (colectomy, coronary artery bypass graft, laparoscopic cholecystectomy, inguinal hernia repair, knee arthroplasty, and spinal fusion).

**Main Outcomes and Measures:**

Readability was measured using previously validated scales (Flesh-Kincaid grade level, Gunning Fog index, the Simple Measure of Gobbledygook, and the Coleman-Liau index). Scores range from 0 to greater than 20 to indicate the years of education required to understand a text. Accuracy and completeness were assessed using a rubric developed with recommendations from LeapFrog, the Joint Commission, and the American College of Surgeons. Both composite and RBA subgroup scores were compared.

**Results:**

The total sample consisted of 36 RBAs, with 1 RBA generated by the LLM-based chatbot and 5 RBAs generated by a surgeon for each of the 6 surgical procedures. The mean (SD) readability score for the LLM-based chatbot RBAs was 12.9 (2.0) vs 15.7 (4.0) for surgeon-generated RBAs (*P* = .10). The mean (SD) composite completeness and accuracy score was lower for surgeons’ RBAs at 1.6 (0.5) than for LLM-based chatbot RBAs at 2.2 (0.4) (*P* < .001). The LLM-based chatbot scores were higher than the surgeon-generated scores for descriptions of the benefits of surgery (2.3 [0.7] vs 1.4 [0.7]; *P* < .001) and alternatives to surgery (2.7 [0.5] vs 1.4 [0.7]; *P* < .001). There was no significant difference in chatbot vs surgeon RBA scores for risks of surgery (1.7 [0.5] vs 1.7 [0.4]; *P* = .38).

**Conclusions and Relevance:**

The findings of this cross-sectional study suggest that despite not being perfect, LLM-based chatbots have the potential to enhance informed consent documentation. If an LLM were embedded in electronic health records in a manner compliant with the Health Insurance Portability and Accountability Act, it could be used to provide personalized risk information while easing documentation burden for physicians.

## Key Points

**Question:**

Can a large language model (LLM)-based chatbot outperform surgeons in generating readable, accurate, and complete procedure-specific risks, benefits, and alternatives (RBAs) for use in informed consent?

**Findings:**

This cross-sectional study of 36 RBAs for 6 commonly performed surgical procedures found that the LLM-based chatbot generated more readable, complete, and accurate consent documentation than the surgeons.

**Meaning:**

These findings indicate that LLM-based chatbots are a promising tool for generating informed consent forms, easing the documentation burden on physicians while providing salient information to patients.

## Teaser

This cross-sectional study compares large language model−based chatbot- vs surgeon-generated information on the risks, benefits, and alternatives of surgical procedures.

## Introduction

Informed consent is a critical component of patient care and is required before any surgical or invasive procedure.^1^ Informed consent is defined as a patient-centered “process of communication” between physician and patient, where the goal is to ensure that the patient understands the risks, benefits, and potential alternatives to a proposed procedure.^2^ This process is key to promoting patient autonomy and safety, and to reducing misunderstanding, mistrust, and patient harm.^3^ However, informed consent is frequently inadequate, in part due to ineffective communication which has been shown to be associated with insufficient shared decision-making.^3,4,5,6,7^ These challenges may be especially pronounced in vulnerable populations.^8,9^

A technique to improve communication and patient comprehension during the informed consent process is the use of electronic consent forms, which have shown to reduce errors, minimize bias, and improve complete documentation of procedure-specific risks, benefits, and alternatives (RBAs) of invasive procedures.^10,11,12^ The use of electronic consent forms may give patients time to review information about the procedure in written form at home, with family or loved ones, and without the time pressure of signing a consent form in the clinic or in the preoperative care unit.^13^

For an electronic consent form to be useful and equitable, the information it provides needs to be readable, accurate, and complete.^14^ Despite national recommendations that they be written for a sixth-grade reading level, studies have shown that informed consent documents are overly complex.^15,16,17,18,19,20,21^ A possible solution to this problem is to leverage artificial intelligence, including generative large language model (LLM)−based chatbots that have been trained to respond to human-generated inquiries.^22^ While relatively nascent in health care, research has shown that LLM-based chatbots can answer patient-generated questions to varying degrees of accuracy.^23,24,25,26^ However, the ability of LLM-based chatbots to enhance informed consent documents remains unknown. This study sought to evaluate RBAs generated by an LLM-based chatbot vs those by surgeons to compare their readability, accuracy, and completeness.

## Methods

This cross-sectional study compared RBAs generated in May 2023 by an LLM-based chatbot (Chat Generative Pretrained Transformer [ChatGPT], model 3.5; OpenAI) with RBAs produced by surgeons for a range of common surgical procedures. The study protocol was deemed exempt by the University of California San Francisco Institutional Review Board and informed consent was waived due to minimal risk to patients. This study was conducted in May 2023 and followed the Strengthening the Reporting of Observational Studies in Epidemiology (STROBE) reporting guideline for observational studies.

### Chatbot Prompt

A multidisciplinary group of surgeons formulated the chatbot prompt: “Explain the risks, benefits, and alternatives of [procedure name] to a patient at a sixth-grade reading level.” The 6 surgical procedures selected were colectomy, coronary artery bypass graft, laparoscopic cholecystectomy, inguinal hernia repair, knee arthroplasty, and spinal fusion because these are common surgical procedures in the US, and they represent a diverse range of surgical specialties.^27^

These questions were inputted into a new profile in the chatbot using the incognito browser mode to mitigate potential bias from other internet activity. ChatGPT is an LLM-based chatbot that responds with text to human-generated questions; it has been shown to perform well on evaluations across multiple domains.^28^ We chose to use model 3.5 because it is distributed free of charge and is therefore most accessible to surgeons across the country. Also, it gave us the ability to enter each prompt into a new instance of the tool, removing any potential bias from prior questions.

### Sample of Surgeon RBAs

At UCSF Health, the operating surgeon documents the RBAs of a surgical procedure in an electronic consent form before the patient reviews and signs it. In 2022, surgeons at UCSF Health underwent mandatory training in informed consent discussions and best practices in documentation; audits are conducted periodically to assess the completeness of documentation and feedback provided to surgeons. For this study, we obtained a random sample of RBAs created by surgeons for each of the included surgical procedures that were signed in May 2023 and were available in the electronic health record (EHR) database. Each consent form was generated by a different surgeon, with 5 unique surgeons per procedure. All surgeons were members of the UCSF Health medical staff (not trainees).

### Measurements of Readability

We evaluated readability using multiple scales, including the previously validated Flesh-Kincaid grade level, Gunning Fog index, the Simple Measure of Gobbledygook score, and the Coleman-Liau index (Table 1).^29^ These measures consider sentence length, word length, and overall text complexity, and provide a US academic grade level for an average student.^30,31^ We calculated readability scores for each scale using an online tool.^32^

**Table 1.** Readability Scales and Interpretation of Scores

Scale	Formula	Historic use	Interpretation
Flesh-Kincaid grade level	(0.39 × [total words/total sentences]) + (11.8 × [total syllables/total words]) – 15.59	US military technical manuals	US grade of education required to understand a text on the first reading
Gunning Fog index	(0.4 × [total words/total sentences]) + (100 × [total complex words/total words])	Public newspapers	6: Sixth grade; 7: seventh grade; 8: eighth grade; 9-12: high school; 13-17: college; >17: college graduate
SMOG index	1.0430 (sqrt [complex words] × [30/number of sentences]) + 3.1291	Health care materials	Years of formal education required to understand a text
Coleman-Liau index	(5.89 × [total characters/total words]) – (0.3 × [total sentences/total words]) – 15.8	US Office of Education textbooks	Years of formal education required to understand a text

Abbreviation: SMOG, Simple Measure of Gobbledygook.

### Measurements of Accuracy and Completeness

To evaluate accuracy and completeness, we developed a scoring system based on recommendations from LeapFrog (VTech Group), the Joint Commission, the American College of Surgeons, and relevant available literature^2,15,23,33^ (Figure). Components of each of the RBAs were evaluated as complete, incomplete, absent, or incorrect and given corresponding scores of 3, 2, 1, and 0, respectively. These scores were combined for a composite score that equally weighted the risks, benefits, alternatives, and overall impression subscores. We used an ensemble scoring strategy, averaging scores across reviewers for each response, as has been used previously in similar studies.^24,34^

**Figure.**
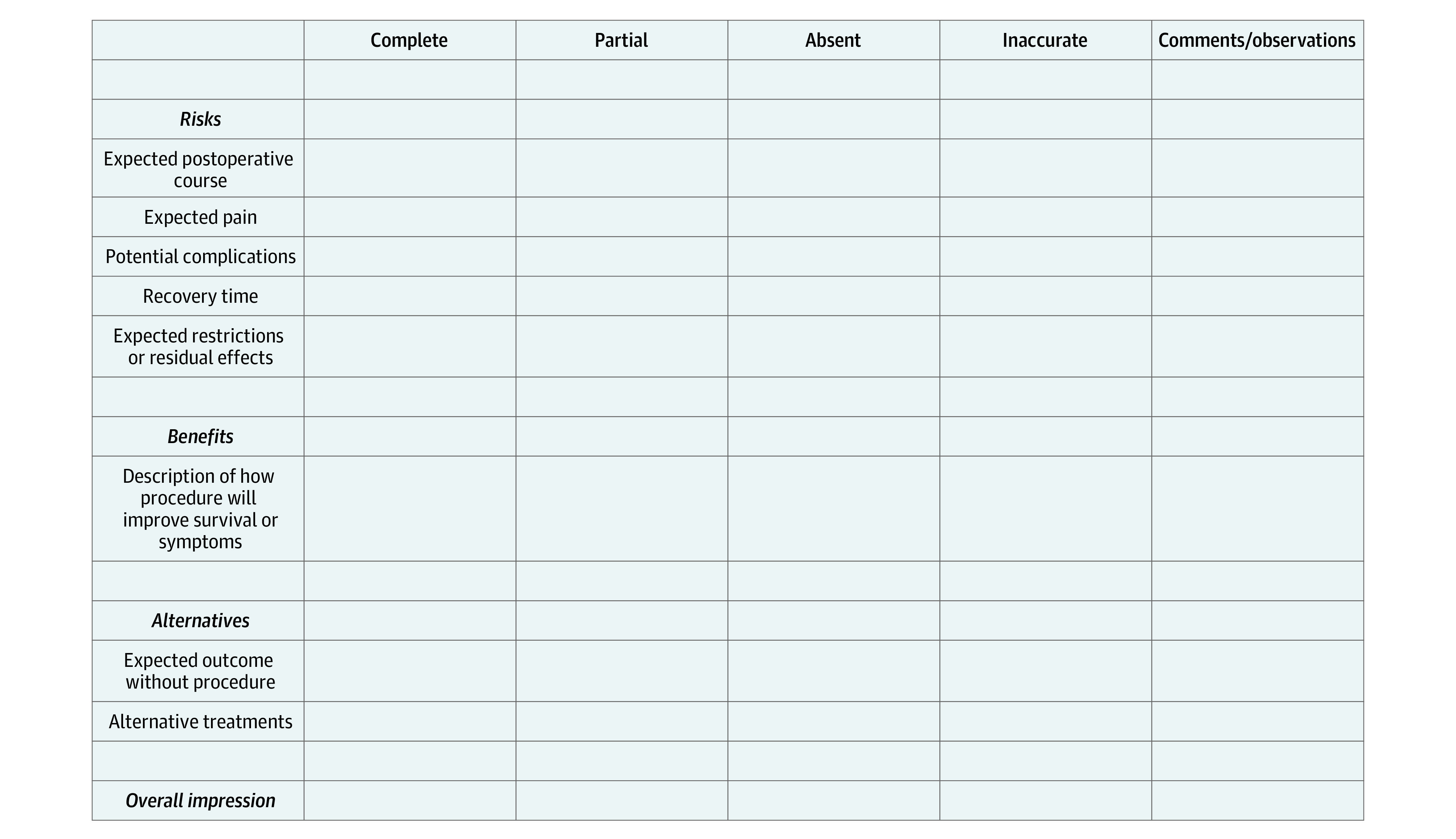
Sample Form for Grading Scale of Informed Consent Risks, Benefits, and Alternatives

A multidisciplinary group of surgeons, including acute care surgery, surgical critical care, vascular surgery, orthopedic surgery, and surgical oncology, reviewed the LLM-based chatbot- and surgeon-generated procedure-specific RBAs for accuracy and completeness using the process described. Reviewers were blinded to the source of the RBA; each response was scored by at least 2 individual reviewers.

### Statistical Analysis

We reported average readability scores for LLM-based chatbot vs surgeon-generated RBAs, both along the individual scales and an average of all scores (as all scales output grade level). We also reported the proportion of RBAs that adhered to important benchmarks (ie, written at a sixth-grade or lower reading level). We reported average accuracy and completeness scores for surgeon-generated and LLM-based chatbot-generated RBAs, as well as the proportion of responses that met key benchmarks (eg, whether responses contained inaccurate information). We compared mean readability, accuracy, and completeness scores of surgeon-generated vs LLM-based chatbot-generated RBAs using Wilcoxon rank-sum tests.

Our primary analyses were comparing the average readability scores for LLM-based chatbot vs surgeon-generated RBAs (averaging all measures and averaging across surgical procedures) and comparing the average accuracy and completeness scores and subscores for LLM-based chatbot vs surgeon-generated RBAs (average across surgical procedures). All other analyses were secondary analyses (including comparisons between procedures) and should be considered exploratory. All hypothesis tests were 2-sided, with an a priori threshold of significance set to *P* < .05. We performed all data analysis in Stata, version 16 (StataCorp).

## Results

### Study Sample

We included 1 LLM-based chatbot- and 5 surgeon-generated RBAs for each of the 6 surgical procedures. Together these yielded 36 responses.

### Readability

The mean readability score for the RBAs generated by ChatGPT was 12.9 (college level) vs 15.7 (college level) for those written by surgeons (Table 2). The LLM-based chatbot-generated RBAs were less complex than the surgeons’ for all 6 surgical procedures, although this difference was not statistically significant when aggregated across surgery types (*P* = .10). The least complex LLM-based chatbot RBA was 10.3 for spine fusion (10th grade) and its most complex was 15.8 for colectomy (college level) (Table 3). The least complex surgeon-generated RBAs averaged 13.6 for spine fusion (college level), and the most complex averaged 17.8 for inguinal hernia (college graduate). No RBAs generated either by the LLM-based chatbot or the surgeons had a readability score of sixth grade or lower, by any readability measures.

**Table 2.** Readability, Accuracy, and Completeness Scores of Informed Consent Documents Generated by Surgeons vs a Large Language Model−Based Chatbot

Area	Score, mean (SD)		
	Surgeons	LLM-based chatbot	*P* value
Readability	15.7 (4.0)	12.9 (2.0)	.10
Accuracy and completeness			
Risks	1.7 (0.5)	1.7 (0.4)	.38
Benefits	1.4 (0.7)	2.3 (0.7)	<.001
Alternatives	1.4 (0.7)	2.7 (0.5)	<.001
Overall impression	1.9 (0.5)	2.3 (0.5)	.001
Composite	1.6 (0.4)	2.2 (0.4)	<.001

**Table 3.** Readability Scores of Risks, Benefits, and Alternatives to Surgery, Generated by Surgeons vs a Large Language Model−Based Chatbot, Stratified by Surgical Procedure

Readability score by scale type, mean (range)											
Laparoscopic cholecystectomy		Inguinal hernia		Colectomy		Coronary artery bypass graft		Knee arthoplasty		Spine fusion	
Surgeon	Chatbot	Surgeon	Chatbot	Surgeon	Chatbot	Surgeon	Chatbot	Surgeon	Chatbot	Surgeon	Chatbot
**Flesh-Kincaid grade level**											
17.7 (6.8-30.7)	12.7	18.1 (10.6-31.7)	11.4	13.8 (8.0-16.1)	15.3	12.1 (9.9-16.4)	9.4	16.8 (11.4-20.9)	12.6	13.0 (9.1-15.6)	8.6
**Gunning Fog index**											
22.7 (11.5-34.4)	15.9	23.4 (16.4-35.4)	16.0	18.0 (11.7-22.0)	19.6	16.2 (14.2-20.0)	13.1	20.5 (14.1-25.1)	17.0	16.5 (10.4-20.8)	12.1
**SMOG index**											
14.0 (7.8-23.3)	11.4	14.2 (10.1-20.7)	11.6	12.6 (8.3-15.2)	14.1	11.1 (9.2-14.4)	9.6	14.4 (10.1-17.1)	12.3	12 (8.0-14.9)	8.6
**Coleman-Liau index**											
15.4 (12.0-21.0)	14.0	15.4 (11.0-22.0)	11.0	15.6 (13.0-18.0)	14.0	16.4 (15.0-18.0)	12.0	13.6 (13.0-15.0)	15.0	13.0 (11.0-14.0)	12.0
**Total, mean (SD)**											
17.5 (3.8)	13.5 (1.9)	17.8 (4.1)	12.5 (2.4)	15.0 (2.4)	15.8 (2.6)	14.0 (2.8)	11.0 (1.8)	16.3 (3.1)	14.2 (2.2)	13.6 (2.0)	10.3 (2.0)

Abbreviation: SMOG, Simple Measure of Gobbledygook.

### Completeness and Accuracy

The mean (SD) composite completeness and accuracy score for all surgeon-generated RBAs was 1.6 (0.5), whereas for LLM-based chatbot-generated, it was 2.2 (0.4) (*P* < .001) (Table 2). When considering each subcategory mean (SD) score, LLM-based chatbot scores were higher than the surgeons’ for description of the benefits of surgery (2.3 [0.7] vs 1.4 [0.7]; *P* < .001), alternatives to surgery (2.7 [0.5] vs 1.4 [0.7]; *P* < .001), and overall impressions (2.3 [0.5] vs 1.9 [0.5]; *P* = .001). There was no significant difference between the LLM-based chatbot- vs surgeon-generated scores for description of the risks of surgery (1.7 [0.5] vs 1.7 [0.4]; *P* = .38).

When considering scores by surgery type, the composite LLM-based chatbot score was higher than the surgeon score for each of the 6 surgical procedures (Table 4). No LLM-based chatbot RBAs were scored as inaccurate on any metric, whereas 3 of 30 surgeon-generated RBAs (10%) were scored as inaccurate on at least 1 metric. In terms of overall impressions, a minority of responses from any source were deemed to be complete (32% of chatbot and 9% of surgeon-generated responses).

**Table 4.** Accuracy and Completeness Scores for Risks, Benefits, and Alternatives to Surgery, Generated by Surgeons vs a Large Language Model−Based Chatbot

Area	Accuracy and completeness score, mean (SD)											
	Laparoscopic cholecystectomy		Inguinal hernia		Colectomy		Coronary artery bypass graft		Knee arthoplasty		Spine fusion	
	Surgeon	Chatbot	Surgeon	Chatbot	Surgeon	Chatbot	Surgeon	Chatbot	Surgeon	Chatbot	Surgeon	Chatbot
Risks	1.4 (0.2)	1.5 (0.3)	1.7 (0.6)	1.8 (0.3)	1.8 (0.5)	1.7 (0.7)	1.3 (0.2)	1.6 (0.3)	2.1 (0.6)	1.7 (0.3)	1.8 (0.5)	1.6 (0.5)
Benefits	1.5 (0.7)	1.3 (0.4)	1.7 (0.8)	2.9 (0.3)	1.4 (0.8)	2.2 (0.6)	1.3 (0.5)	2.2 (0.4)	1.5 (0.5)	2.8 (0.3)	1.5 (0.7)	2.6 (0.5)
Alternatives	1.4 (0.7)	2.4 (0.9)	1.5 (0.9)	2.8 (0.5)	1.6 (0.9)	2.8 (0.4)	1.4 (0.7)	2.6 (0.5)	1.3 (0.6)	3.0 (0)	1.2 (0.4)	2.8 (0.4)
Overall impression	1.9 (0.3)	2.3 (0.5)	1.9 (0.4)	2.7 (0.6)	2.0 (0.6)	2.4 (0.5)	1.6 (0.6)	2.3 (0.7)	2.2 (0.4)	2.0 (0)	2.1 (0.5)	2.3 (0.5)
Composite	1.6 (0.3)	1.9 (0.4)	1.7 (0.5)	2.5 (0.3)	1.6 (0.6)	2.3 (0.5)	1.4 (0.4)	2.2 (0.3)	1.8 (0.5)	2.4 (0.1)	1.6 (0.4)	2.3 (0.4)

## Discussion

Much has been written about the potential of artificial intelligence and LLMs to alter the practice of medicine.^35,36^ However, few studies to date have provided data on reliability, completeness, and accuracy of output of LLMs in surgical contexts, especially those related to operative consents. In this study comparing LLM-based chatbot- vs surgeon-generated informed consents for common surgical procedures, we found that the chatbot RBAs were more readable, complete, and accurate than those produced by surgeons.

In terms of readability, every surgeon and chatbot-generated RBA was more complex than the recommended sixth-grade reading level. This finding is consistent with many other studies reporting that informed consent documentation is too complex.^16,17,19,20,21^ However, the chatbot-generated RBAs did trend toward being less complex compared with surgeons’ RBAs for all 6 included surgical procedures. Given its interactivity, users have the ability to ask the LLM-based chatbot to make its response even more simple which may yield responses at the target reading level.^37^ However, the tool did not reliably generate RBAs for common procedures at the sixth-grade reading level even when prompted to do so.

When compared with surgeon-generated RBAs, LLM-based chatbot-generated RBAs had better scores for completeness and accuracy for every surgical procedure specified per our established study rubric. This difference was primarily driven by the LLM-based chatbot descriptions of the benefits and alternatives to surgery, given that there were no significant differences in the descriptions of surgical risks between the 2 sources. The reviewers infrequently assessed the consents as being inaccurate and the only consents with inaccurate elements in the study sample were generated by surgeons. However, caution should still be used when incorporating LLM-based chatbot responses into informed consent documents, in which reliability is critical, given that these models have been shown to generate plausible but incorrect responses.^28^ Given this dynamic, at least for the time being, physicians should have to review and edit informed consent documentation generated by LLM-based chatbots. However, this approach still may be simpler, less time-consuming, and more accurate than generating informed consent de novo each time. Future studies should compare informed consent documentation generated by LLM-based chatbots and reviewed by physicians vs those generated by surgeons alone.

Some surgeon-generated RBAs described a conversation with the patient detailing the risks, benefits, and alternatives to surgery rather than documenting them explicitly. Although a thorough conversation between surgeon and patient is essential for informed consent to occur, failing to also document the salient risks, benefits, and alternatives in the consent form deprives the patient of additional time to review the information at home, with family, caregivers, or other resources available. This opportunity for review is critical considering that prior research has shown that patients immediately forget 40% to 80% of medical information provided by clinicians and almost half of the information that is remembered is incorrect.^38^ Furthermore, documenting that a conversation occurred, rather than describing the information relayed in the conversation, may not provide appropriate protection in the event of litigation.

Additional studies should incorporate patient perspectives and opinions on the adequacy of LLM-based chatbot-generated informed consent documentation to build on this work. Ultimately, if an LLM is embedded in the EHR in a manner compliant with the Health Insurance Portability and Accountability Act, we anticipate it could be used to provide personalized risk language based on disease severity and underlying conditions.^39^ Together, this would allow for the adoption of more transparent sharing of complication risks with patients as part of the informed consent process. Finally, we expect that LLMs will allow for additional training examples of consent language (as opposed to zero-shot training used in this study) for continuously improved results that are better optimized for automated generation of more personalized informed consent language.

### Limitations

This study had certain limitations. First, we chose use version 3.5 of the chatbot because it is accessible without a paid subscription. Given that model 4.0 has already been shown to outperform model 3.5 on various professional and academic benchmarks,^28^ we anticipate that the gaps we demonstrated will widen as LLMs continue to evolve and improve. Second, our study rubric incorporated the documentation of expectations (expected pain, postoperative recovery, and restrictions or residual effects). Some surgeons may not have known to include this information in their documentation of risks. However, setting patient expectations has been shown to be an essential part of patient-physician trust and relationships.^40^ There were no significant differences in ChatGPT- vs surgeon-generated RBAs along these specific metrics, suggesting that the rubric was not unfairly penalizing to surgeons. Third, our study evaluated the LLM-based chatbot -generated RBAs without physician alteration. This is unlikely to occur in practice. Further study should be undertaken to evaluate consent documents created by LLM-based chatbots and surgeons working together, which is a more realistic application of LLMs in the health care settings. We conducted this study at a single quaternary care center (UCSF Health) where the practice of surgeons documenting information in the EHR may differ from that of other institutions. In general, surgical informed consent discussions and documentation have not been actively taught as part of surgical training and are far from standardized. The introduction of artificial intelligence in this area may be an opportunity to recognize the importance of this gap and develop new training paradigms for surgeons.

## Conclusions

This cross-sectional study found that that RBAs generated by an LLM-based chatbot were more readable, accurate, and complete than RBAs generated by surgeons for 6 common surgical procedures. Large language model−based chatbots are a promising tool that could ease the documentation burden on physicians while providing salient information preoperatively to patients in language that they can understand.

## Notes

Supplement.
Data Sharing Statement
