# Supplementary material for: Large Language Model−Based Chatbot vs Surgeon-Generated Informed Consent Documentation for Common Procedures
Source: JAMA Netw Open. 2023 Oct 9;6(10):e2336997. doi: 10.1001/jamanetworkopen.2023.36997 (PMC10562939; doi:10.1001/jamanetworkopen.2023.36997)
Supplement: Supplement. — Data Sharing Statement [file jamanetwopen-e2336997-s001.pdf]

Data Sharing Statement

Decker. Large Language Model-Based Chatbot vs Surgeon-Generated Informed Consent Documentation for Common Procedures. *JAMA Netw Open*. Published October 09, 2023. doi:10.1001/jamanetworkopen.2023.36997

Data
Data available: Yes
Data types: Deidentified participant data
How to access data: Hannah.decker@ucsf.edu
When available: With publication

Supporting Documents
Document types: None

Additional Information
Who can access the data: Researchers for whom a project has been approved.
Types of analyses: Approved research.
Mechanisms of data availability: After approval of a proposal.
